# Independent predictors of non-suicidal self-injury among adolescents: a logistic regression model development study based on coping styles

**DOI:** 10.3389/fpsyg.2026.1930200

**Published:** 2026-10-29

**Authors:** Qiuyu Yuan, Chenlu Yao, Changping Zhai, Xiaoqin Zhou

**Affiliations:** 1 Anhui Province Veterans Hospital, Bengbu, Anhui, China; 2 School of Mental Health, Bengbu Medical University, Bengbu, Anhui, China; 3 Department of Psychology and Sleep Medicine, The Second Affiliated Hospital of Anhui Medical University, Hefei, Anhui, China

**Keywords:** adolescent, nomogram, non-suicidal self-injury, problem-solving, self-blame

## Abstract

**Objective:**

Non-suicidal self-injury (NSSI) is an urgent and substantial public health concern among adolescents. This study is aimed at identifying the independent psychological predictors of NSSI and developing and validating a logistic regression-based risk discrimination model focused on coping styles.

**Methods:**

A cross-sectional study involving 206 adolescents (104 with NSSI and 102 healthy controls) was conducted. All the participants provided general demographic data, and completed the Experiences in Close Relationships Inventory (ECR), the Childhood Trauma Questionnaire-Short Form Version (CTQ-SF), and the Coping Styles Questionnaire (CSQ). Univariate and multivariate logistic regression analyses were performed to identify the independent predictors of NSSI. A nomogram was constructed in accordance with the final multivariate model. Model performance was evaluated using receiver operating characteristic (ROC) curves, calibration plots, the Hosmer-Lemeshow (HL) test, and a decision curve analysis (DCA).

**Results:**

Multivariate logistic regression revealed that a higher level of self-blame (OR = 1.062; 95% CI: 1.023–1.102; *P* = 0.002) was an independent risk factor, whereas a better problem-solving capacity (OR = 0.911; 95% CI: 0.867–0.957; *P* < 0.001) was an independent protective factor for adolescents with NSSI. Attachment dimensions and childhood trauma did not retain independent significance after adjustment. The nomogram showed excellent discrimination (AUC = 0.951, 95% CI: 0.922–0.980), good calibration (HL test *P* > 0.05), and superior clinical net benefit across a wide threshold (0.01–0.99).

**Conclusion:**

Targeting self-blame and enhancing problem-solving skills may be effective styles for the prevention of adolescent NSSI. As a preliminary finding, the nomogram developed in our study is a useful tool for exploratory differentiation.

## 1 Introduction

Individuals who do not intend to commit suicide but who, with specific purpose, act consciously to harm their own body tissue–including by cuts, burns, scratches, and impacts–are defined as individuals with non-suicidal self-injury (NSSI; Cipriano et al., 2017; Klonsky et al., 2014). NSSI has emerged as a major public health concern among children and adolescents, posing persistent societal challenges. Worldwide, an estimated minimum of 14 million self-harm episodes occur every year, for an incidence rate of 60 cases per every 100,000 individuals (Moran et al., 2024). The lifetime prevalence of NSSI among Chinese adolescents is 24.7% (Qu et al., 2023). Adolescents who engage in NSSI face increased risks of multiple adverse outcomes, including poor academic performance, difficulties with key developmental tasks, mental disorders, and suicidal behaviors, which in turn exacerbate the burden on families and society (Gulbas et al., 2015; Hamza and Willoughby, 2016; Kiekens et al., 2016; Robinson et al., 2019).

Non-suicidal self-injury is not singular but rather a result of the interaction of various psychosocial factors. That the emotions and behaviors of adolescents are likely related to the characteristic ways that they relate to early intimacy from their parents is widely accepted. On the basis of the initial description of Bowlby, the concept of “attachment” is proposed; that is, the repeated communication experience of infants and young children with their caregivers was internalized into activity models that were then gradually integrated into an overall the personality structure to influence emotional and behavioral development on the basis of cognitive functions (Bowlby, 1978, 1982). Children with anxious–ambivalent attachment styles tend to employ an “overactivation” strategy, aimed at obtaining their caregivers’ attention and care. Regardless of their age, anxiously attached individuals typically have lower levels of self-evaluations but higher evaluations of others. They persistently maintain interpersonal closeness and strive to ensure that they can receive support (Ainsworth et al., 1978; Cole-Detke and Kobak, 1996). In contrast, individuals with avoidant attachment styles have high levels of self-evaluations and low evaluations of others. They have an overreliance on their own abilities, have difficulty adapting to emotional intimacy in relationships, and often neglect their own emotional needs (Cole-Detke and Kobak, 1996; Mikulincer and Shaver, 2012). Studies have shown that in clinical samples of adolescents, insecure attachment is correlated with non-suicidal self-injurious behavior (Woo et al., 2022). A widely accepted theoretical assumption posits that these effects are achieved through the joint mediation of interpersonal relationship regulation and individual internal emotional regulation (Nock, 2010). In terms of specific mechanisms, individuals with anxious–ambivalent attachment tend to use NSSI behaviors to express their overly activated emotions, thereby obtaining more attention and contact from their object of attachment (Adam et al., 1996; Michelson and Bhugra, 2012). This behavior is in sharp contrast to that of avoidant attachment individuals: who use self-injurious behaviors to downregulate their emotional responses with an the aim of reducing the support received from their object of attachment as well as the level of contact (Tasca et al., 2009).

Furthermore, one theory has suggested that adverse experiences that occurred during childhood may hinder the formation of good emotional regulation abilities among individuals, leaving them more prone to intense emotional pain. Adverse childhood experiences may increase the risk of NSSI. Cumulative exposure to different forms of childhood adversity (such as physical, sexual, and emotional abuse) is associated with an increased risk of NSSI (He et al., 2026). Experiences such as emotional neglect, family conflicts, or unstable family environments may weaken individuals’ social support systems and coping resources, thereby increasing their likelihood of engaging in self-harm behavior (Alkema et al., 2024). Negative emotions and emotion-oriented coping styles play a mediating role in the influence of parent–child attachment on self-harm behaviors (Tao et al., 2020). Additionally, in this context, self-harm behaviors may evolve into a maladaptive coping strategy for the alleviation of unbearable emotions or psychological pain (Chen et al., 2022; Cheng et al., 2023).

Mature coping styles affect an individual’s mental health and interpersonal functioning, particularly among children and adolescents with a history of childhood trauma. This understanding has clear guiding significance for their treatment and intervention (Shapiro and Levendosky, 1999). Further analyses by some scholars has revealed that adolescents who experience a lower sense of security toward their parents in early childhood are more likely to adopt emotion-oriented coping styles in adulthood, and this coping style plays a partial mediating role between adolescents’attachment insecurity toward their parents and their attachment anxiety (Pascuzzo et al., 2013). Notably, even in research conducted within the “behavioral addiction” field, coping styles have been demonstrated to have both mediating effects and regulatory functions in the relationship between attachment and video game addiction as well as that between attachment and problematic internet use (Estevez et al., 2019). Collectively, this evidence indicates that coping styles are important influencing variables on adolescent NSSI behaviors. Therefore, the early identification of high-risk factors for self-harm and timely intervention among adolescents can reduce the incidence of adverse events associated with self-injury and suicide (Kiekens et al., 2023).

In the literature, the mediating effects of diverse psychosocial variables on the development of non-suicidal self-injury among adolescents has been often explored. In contrast to the statistical analysis methods used in traditional medical research, the use of clinical discrimination models can facilitate the integration of multiple variables in assessing the probability of an individual presenting with a specific condition or exhibiting a certain outcome status (Cheng et al., 2022). Nevertheless, all the aforementioned variables have rarely been incorporated into a single risk discrimination model to generate visualized models for clinical guidance; for student populations in particular, the number of such models remains limited. To address this research gap, the hypothesis that attachment anxiety/avoidance, specific coping styles (such as self-blame, avoidance, and fantasy), and childhood trauma are independent predictors of adolescent self-harm is advanced in this study. The goal of this study was (1) to determine the independent risk factors for self-harm by comparing self-harming adolescents with healthy controls and (2) to construct and validate a risk discrimination model based on logistic regression, which can be used to estimate the probability of an individual engaging in self-harm.

## 2 Materials and methods

### 2.1 Recruitment

We recruited adolescents who met the NSSI diagnostic criteria from among inpatients in psychosomatic wards and outpatients in psychological counseling at Chaohu Hospital of Anhui Medical University, between September and December 2019. All the participants as students were enrolled in middle schools in Chaohu city. Before the recruitment interviews, we considered the adolescents’ self-reported histories of NSSI during the proceeding year and supplementary material from parents who had observed pathological behaviors in the adolescents. Additionally, we observed the scars of the adolescents (mild or moderate scratches or cutting on the forearms and wrists) over a period of 2 weeks to identify adolescents with NSSI. This study involving human participants was reviewed and approved by the Ethics Committee of Chaohu Hospital of Anhui Medical University (No. 2020 11-kyxm-03). Prior to administering the questionnaire, informed written consent was obtained from all the adolescents and the legal guardians (parents or grandparents) of the minors (<18 years old) who participated in the research. Verbal consent was obtained from each participant.

Moreover, the healthy controls included students from Chaohu No. 2 Middle School. Prior to the investigation, an investigator gave a consistent and accurate explanation to the subjects regarding the requirements for completing the questionnaire. During the investigation, all the adolescents wrote and answered the questions independently and anonymously (as an anonymous setting is more conducive to the disclosure of their inner voice). Within the specified time, we collected the questionnaires after the adolescent participants had completed them.

### 2.2 Participants

#### 2.2.1 NSSI group

The inclusion criteria were as follows: the participants had to be adolescents with NSSI who were aged 12–18 and had the ability to understand the reading material in Mandarin. They had to be of Han ethnicity, right-handed, and most importantly, they had to meet the DSM-5 diagnostic criteria of NSSI-A (frequency and course) as diagnosed by a chief psychiatrist (Battle, 2013). In the current study, it was stipulated that any included individual must have reported at least 5 days of NSSI during the previous year. Moreover, to meet the criteria for the NSSI-B, as determined by the participant interviews, they must be free of suicidal intent, and they must regard NSSI as a means of reducing emotional pain. The exclusion criteria were as follows: the participants must not have serious physical or mental diseases (meeting the DSM-5 diagnostic criteria for organic psychiatric disorders, schizophrenia, and bipolar disorder), and they must not show a refusal to cooperate with the investigation.

Interestingly, in this study, the pathological impulsive behavior demonstrated by those in the NSSI group was presented only by sharp cuts or scratches on the wrists/forearms. The fact that the suggestions on how to select minimum frequency diagnostic criteria for NSSI ensure the validity of major targets as much as possible remains undisputed. To better approximate the proposed diagnostic criteria in the DSM-5, the current study included individuals who self-reported at least 5 days of NSSI over the previous year and at least 4 days of NSSI during the previous month. First, 80%–90% of the clinicians and researchers selected from the American Psychological Association division supported the diagnostic item NSSI-A in the DSM-5 as the characteristic statement indicating NSSI (Lengel and Mullins-Sweatt, 2013). Second, it was also based on feedback from clinicians and researchers in the field of NSSI, which suggested that requiring 4 days of NSSI behaviors in the previous month might enhance clinical utility (Ammerman et al., 2021); Maura Manca previously reported a more clinically-compromised profile for repetitive (rather than occasional) self-injurers (Manca et al., 2014), which should be clinically significant over the short term. All adolescents with NSSI were enrolled shortly after their self-injury episodes, which enables a reliable assessment of the associations between NSSI and its relevant psychosocial factors. Thus, the level of recall bias in the self-reported data was minimal.

#### 2.2.2 Healthy controls

The inclusion criteria were as follows: subjects must be aged 12–18, have the ability to understand the reading material in Mandarin, be of the Han ethnicity, and be right-handed, Each of the participants in the healthy controls were evaluated for mental status by the same chief psychiatrist. The exclusion criteria were as follows: subjects must not have serious physical or mental diseases (meeting the DSM-5 diagnostic criteria for organic psychiatric disorders, schizophrenia, and bipolar disorder), exhibit present or past NSSI behaviors, or show a refusal to cooperate with the investigation.

Finally, a total of 214 questionnaires were distributed. Eight of them were incomplete, yielding 206 valid responses, which included 104 adolescents with NSSI (15 males, 89 females) and 102 healthy adolescents (50 males, 52 females). Given the relatively low proportion of missing values and the exploratory nature of the present prediction-model study, we adopted the complete-case analysis method for all analyses, so only those participants with complete information for all the variables were included in final analysis.

### 2.3 Assessments

#### 2.3.1 Self-made demographic information scale

This information included the participants’ data on age, sex, sibling status, parental marital status, parental education level and family economic status.

#### 2.3.2 Experiences in Close Relationships Inventory (ECR)

The ECR compiled by Brennan, Clark and Shaver was used to assess the attachment dimensions of the participants (Brennan and Shaver, 1998). The translated and revised Chinese version of the Experiences in Close Relationships Inventory also has a relatively ideal retest reliability and internal consistency (Zhang et al., 2022). There are two subscales, i.e., attachment avoidance and attachment anxiety, with 18 questions with responses ranging from 1 to 7 points, which indicates a range from “strongly disagree” to “strongly agree.” The ECR has proven to be the standard questionnaire for measuring and defining attachment. Previous studies have fully demonstrated that the self-reported attachment dimensions (attachment avoidance and attachment anxiety) enables more sensitive measurements of subtle differences, which is helpful for accurate assessment (Collins, 1996). We decided to assess the two attachment dimensions, a process which uncovered more unambiguity among the assessments, and to reject the concept of attachment insecurity as a blend of avoidance and anxiety dimensions, which avoided any additional variability among the complex groupings.

#### 2.3.3 Childhood Trauma Questionnaire Short-Form Version (CTQ-SF)

The CTQ-SF is a validated 28-item retrospective self-report instrument created by Bernstein et al. (1994) to measure five types of childhood maltreatment: emotional abuse, physical abuse, sexual abuse, emotional neglect, and physical neglect (Bernstein et al., 1994, 1997). Participants respond to each item on a 5-point Likert scale from 1 (Never True) to 5 (Very Often True). Given its sound reliability and cross-cultural validity, the CTQ-SF has been universally adopted in clinical screening and empirical research to quantify the severity of early traumatic experiences and examine their links with mental health disorders (Bernstein et al., 2003).

#### 2.3.4 Coping Style Questionnaire (CSQ)

One of the standardized tools used for studying the coping styles of different groups is the questionnaire on coping styles compiled by Xiao and Xu, which includes the following six subscales: problem solving, rationalization, self-blame, help-seeking, fantasy and withdrawal. All 62 questions are scored from the two options of “likely to choose” or “unlikely to choose.” The combination of different salience scores according to the subscales revealed the styles used to cope with unique pressures. The use of the CSQ in group studies has generally proven to be reliable and stable (Xiao Jihua, 1996).

### 2.4 Statistical analysis

The data were analyzed using SPSS 27.0 and R software (version 4.2.1). The continuous variables are presented as the mean ± SD or median (IQR), and the categorical variables are presented as frequencies and percentages. Group comparisons were performed using independent *t*-tests, Mann-Whitney U tests, or Pearson’s chi-square tests. Specifically, factor scores on the Coping Style Questionnaire fell within the range of 0.00–1.00 and are reported to two decimal places. These factor scores were multiplied by 100 to enable a regression coefficient interpretation, such that the regression coefficient could reflect the change in log-odds that corresponds to each 100-unit increment of the CSQ factors. Original scores are presented in the baseline characteristics table (Table 1). Univariate logistic regression was used to screen potential factors. All variables for which *P* was < 0.05 according to the univariate analysis were entered simultaneously (Enter method) into a multivariate logistic regression model. Multicollinearity was assessed via the variance inflation factor (VIF > 5 indicated collinearity). The independent factors for which *P* was < 0.05 in the final model were used to construct a nomogram. Model discrimination was evaluated via the ROC curve (AUC with 95% CI), and calibration was evaluated via the calibration curve and HL (*P* > 0.05 indicated good calibration). Decision curve analysis (DCA) was used to assess the clinical net benefit. The nomogram, calibration curve, and DCA were plotted using Free Statistics software (version 1.7.1). There were no *a priori* sample size calculations performed. A two-sided *P* < 0.05 indicates statistical significance. The data were analyzed from March to May 2026.

**TABLE 1:** Demographic and clinical characteristics of adolescents with nonsuicidal self-injury (NSSI) and healthy controls.

Variables	Total (*n* = 206)	NSSI (*n* = 104)	Healthy controls (*n* = 102)	P	t/*χ*^2^
Age	15.5 ± 1.2	15.5 ± 1.4	15.6 ± 0.8	0.436	0.61
Sex				<0.001	28.54
Male	65 (31.6)	15 (14.4)	50 (49)		
Female	141 (68.4)	89 (85.6)	52 (51)		
Sibling status				0.071	3.27
Yes	98 (47.6)	43 (41.3)	55 (53.9)		
No	108 (52.4)	61 (58.7)	47 (46.1)		
Parental marital status				<0.001	11.58
Married	174 (84.5)	79 (76)	95 (93.1)		
Divorcee	32 (15.5)	25 (24)	7 (6.9)		
Parental education level				0.062	3.49
Low^a^	25 (12.1)	17 (16.3)	8 (7.8)		
High^b^	181 (87.9)	87 (83.7)	94 (92.2)		
Family economic status				<0.001	12.06
Average or above	162 (78.6)	92 (88.5)	70 (68.6)		
Poor	44 (21.4)	12 (11.5)	32 (31.4)		
ECR					
Attachment avoidance	4.2 ± 1.2	5.0 ± 1.0	3.4 ± 0.9	<0.001	130.32
Attachment anxiety	3.1 ± 1.0	3.6 ± 1.0	2.6 ± 0.7	<0.001	61.09
CTQ-SF					
Physical neglect	9.7 ± 3.2	10.8 ± 3.2	8.7 ± 2.7	<0.001	25.45
Emotional neglect	14.6 ± 5.7	17.3 ± 5.2	11.8 ± 4.9	<0.001	61.41
Physical abuse	7.1 ± 3.3	8.1 ± 4.0	6.1 ± 2.1	<0.001	21.68
Emotional abuse	10.2 ± 5.1	13.1 ± 5.1	7.2 ± 3.0	<0.001	100.26
Sexual abuse	5.6 ± 1.9	6.1 ± 2.5	5.2 ± 0.6	<0.001	13.44
CSQ					
Problem-solving	0.6 ± 0.3	0.4 ± 0.2	0.8 ± 0.2	<0.001	174.54
Rationalization	0.4 ± 0.2	0.4 ± 0.2	0.4 ± 0.2	0.081	3.07
Self-blame	0.5 ± 0.3	0.7 ± 0.2	0.4 ± 0.2	<0.001	195.48
Help-seeking	0.5 ± 0.3	0.3 ± 0.2	0.6 ± 0.2	<0.001	119.12
Fantasy	0.5 ± 0.2	0.6 ± 0.2	0.5 ± 0.2	<0.001	16.71
Withdrawal	0.5 ± 0.2	0.6 ± 0.2	0.4 ± 0.2	<0.001	29.35

Data are presented as mean ± standard deviation for continuous variables, and as *n* (%) for categorical variables. *P*-values were calculated using independent samples *t*-test for continuous variables and Pearson’s chi-square test for categorical variables.

Low^a^ parental education: neither parent completed 9 years of compulsory education.

High^b^ parental education: at least one parent completed 9 years of compulsory education.

## 3 Results

### 3.1 Comparison of the general information between the two groups

A total of 206 adolescents were enrolled in this study, including 102 healthy controls and 104 adolescents in the NSSI group. No statistically significant differences were observed between the two groups in terms of age, sibling status, or parental educational level (*P* > 0.05). However, significant differences were found in terms of gender, parental marital status, and family economic status (*P* < 0.05). First, in the NSSI group, the average age of the adolescents was 15.5 (with a standard deviation of 1.4) years, and the proportion of girls was observably high (85.6%). The reasons for the “girl risk” phenomenon are explained in the Discussion. The detailed results are presented in Table 1.

### 3.2 Comparison of disease-related characteristics between the two groups

The two groups showed statistically significant differences in regard to attachment avoidance, attachment anxiety, coping styles (problem-solving, self-blame, help-seeking, fantasy, and withdrawal), and all of the subscales of childhood trauma (*P* < 0.05). No significant difference was found in the coping style of rationalization between the two groups (*P* > 0.05). The detailed data are also shown in Table 1.

### 3.3 Univariate logistic regression analysis

Univariate logistic regression analysis was conducted with the presence of NSSI behavior in adolescents as the dependent variable (NSSI = 1, no NSSI = 0). The variables that were significantly different between the two groups were entered as independent variables. The original values were used for the continuous variables, and the categorical variables were coded as follows: gender, male = 0, female = 1; only-child status, no = 0, yes = 1; parental marital status, divorced = 0, married = 1; parental education level, low (neither parent completed 9 years of compulsory education) = 0, high (at least one parent completed 9 years of compulsory education) = 1; and family economic status, poor = 0, average or above = 1. The results revealed that gender, parental marital status, family economic status, attachment avoidance, attachment anxiety, problem-solving, self-blame, help-seeking, fantasy, withdrawal, and all the CTQ factors significantly associated with NSSI (*P* < 0.05). No significant associations were found between NSSI and age, sibling status, parental educational level, or rationalization coping style (*P* > 0.05). The detailed data are presented in Table 2.

**TABLE 2:** Univariate logistic regression analysis of adolescents with nonsuicidal self-injury (NSSI).

Variables	β	SE	Wald *χ*^2^	OR	95% CI	P
Age	−0.094	0.121	0.612	0.910	(0.718–1.153)	0.434
Sex						
Male	1.000 (Ref)					
Female	1.741	0.342	25.888	5.705	(2.917–11.158)	<0.001
Sibling status						
No	1.000 (Ref)					
Yes	0.507	0.281	3.248	1.660	(0.957, 2.881)	0.072
Parental marital status						
Divorced	1.000 (Ref)					
Married	−1.457	0.454	10.309	0.233	(0.096–0.567)	0.001
Parental education level						
Low^a^	1.000 (Ref)					
High^b^	−0.831	0.454	3.354	0.436	(0.179–1.060)	0.067
Family economic status						
Poor	1.000 (Ref)					
Average or above	−1.254	0.374	11.255	0.285	(0.137–0.594)	0.001
ECR						
Attachment anxiety	1.245	0.201	38.322	3.472	(2.341–5.149)	<0.001
Attachment avoidance	1.495	0.204	53.567	4.459	(2.988–6.655)	<0.001
CTQ-SF						
Physical neglect	0.238	0.052	20.599	1.268	(1.145–1.406)	<0.001
Emotional neglect	0.199	0.031	40.350	1.220	(1.148–1.298)	<0.001
Physical abuse	0.257	0.065	15.501	1.291	(1.138–1.469)	<0.001
Emotional abuse	0.342	0.049	48.314	1.407	(1.278–1.550)	<0.001
Sexual abuse	0.442	0.142	8.846	1.524	(1.155–2.013)	0.003
CSQ						
Problem solving (×100)	−0.081	0.011	58.532	0.922	(0.903–0.941)	<0.001
Rationalization (×100)	0.014	0.008	3.013	1.014	(0.998–1.031)	0.083
Self-blame (×100)	0.079	0.010	63.103	1.082	(1.061–1.103)	<0.001
Help-seeking (×100)	−0.061	0.008	54.352	0.941	(0.926–0.956)	<0.001
Fantasy (×100)	0.030	0.008	14.546	1.030	(1.015–1.046)	<0.001
Withdrawal (×100)	0.039	0.008	23.395	1.039	(1.023–1.056)	<0.001

OR, odds ratio; 95% CI, 95% confidence interval; β, regression coefficient; SE, standard error. All six CSQ subscale scores were multiplied by 100 prior to the analysis to facilitate the interpretation of odds ratios.

Low^a^ parental education: neither parent completed 9 years of compulsory education.

High^b^ parental education: at least one parent completed 9 years of compulsory education.

### 3.4 Multivariate logistic regression analysis

The results revealed that higher levels of self-blame (OR = 1.062, 95% CI: 1.023–1.102, *P* = 0.002) were an independent risk factor for the presence of non-suicidal self-injury (NSSI) behaviors among the sample adolescents, whereas better problem-solving ability (OR = 0.911, 95% CI: 0.867–0.957, *P* < 0.001) was an independent protective factor. Gender, parental marital status, family economic status, attachment avoidance, attachment anxiety, all the subtypes of childhood trauma, help-seeking, fantasy, and withdrawal were not independently associated with NSSI after adjustment (all *P* > 0.05). The detailed results of the data analysis are presented in Table 3.

**TABLE 3:** Multivariate logistic regression analysis of factors associated with NSSI among adolescents.

Variables	β	SE	Wald *χ*^2^	OR	95% CI	P
Constant	−5.151	3.756	1.880	0.006		0.170
Sex						
Male						
Female	1.640	0.866	3.591	5.157	(0.945–28.135)	0.058
Parental marital status						
Divorced						
Married	−0.556	1.170	0.226	0.574	(0.058–5.679)	0.635
Family economic status						
Average or above						
Poor	0.948	0.919	1.065	2.581	(0.426–15.623)	0.302
ECR						
Attachment avoidance	0.631	0.397	2.530	1.879	(0.864–4.088)	0.112
Attachment anxiety	0.410	0.519	0.625	1.507	(0.545–4.167)	0.429
CTQ–SF						
Physical neglect	0.222	0.176	1.581	1.248	(0.883–1.763)	0.209
Emotional neglect	−0.071	0.102	0.476	0.932	(0.763–1.139)	0.490
Physical abuse	0.178	0.178	0.636	1.194	(0.772–1.848)	0.425
Emotional abuse	0.302	0.178	2.877	1.353	(0.954–1.918)	0.090
Sexual abuse	−0.254	0.336	0.574	0.775	(0.402–1.497)	0.449
CSQ						
Problem solving (×100)	−0.093	0.025	13.858	0.911	(0.867–0.957)	<0.001
Self-blame (×100)	0.060	0.019	10.078	1.062	(1.023–1.102)	0.002
Help-seeking (×100)	−0.031	0.019	2.660	0.970	(0.935–1.006)	0.103
Fantasy (×100)	0.020	0.021	0.881	1.020	(0.979–1.063)	0.348
Withdrawal (×100)	−0.021	0.021	1.078	0.979	(0.940–1.019)	0.299

OR, odds ratio; 95% CI, 95% confidence interval; β, regression coefficient; SE, standard error. Variables with *P* < 0.05 in the univariate analysis were entered into the multivariate model using the enter method. All six CSQ subscale scores were multiplied by 100 prior to the analysis to facilitate the interpretation of odds ratios.

### 3.5 Model construction and evaluation

On the basis of two independent predictors identified through multivariate logistic regression, namely, problem-solving ability and self-blame–a nomogram was developed to discriminate non-suicidal self-injury (NSSI) among adolescents (Figure 1). The ROC curve showed an area under the curve (AUC) of 0.951 (95% CI: 0.922–0.980), which indicates that the model has excellent discriminatory power (Figure 2). Internal validation using 1,000 bootstrap resamples yielded a corrected Dxy of 0.900 and an optimism bias of 0.003, which confirms that the model exhibits low overfitting. The HL test results were not statistically significant (*P* > 0.05), and the bias-corrected calibration curve was closely aligned with the ideal line (mean absolute error = 0.007, Emax = 0.007; corrected intercept = −0.006, slope = 0.973), which indicated good model calibration (Figure 3). Decision curve analysis (DCA) revealed that, within the threshold probability range of 0.01–0.99, the net benefit of this model was consistently greater than that of the “full intervention” or “no intervention” styles (Figure 4). The optimal cutoff value (Jordaan index) was 0.462. At this threshold, the model’s accuracy was 89.32%, its sensitivity was 87.56%, specificity was 90.14%, positive predictive value was 88.79%, and its negative predictive value was 89.85%. The bias-corrected Brier score was 0.082, and R^2^ was 0.752, indicating a low level of prediction error and strong explanatory power.

**FIGURE 1:**
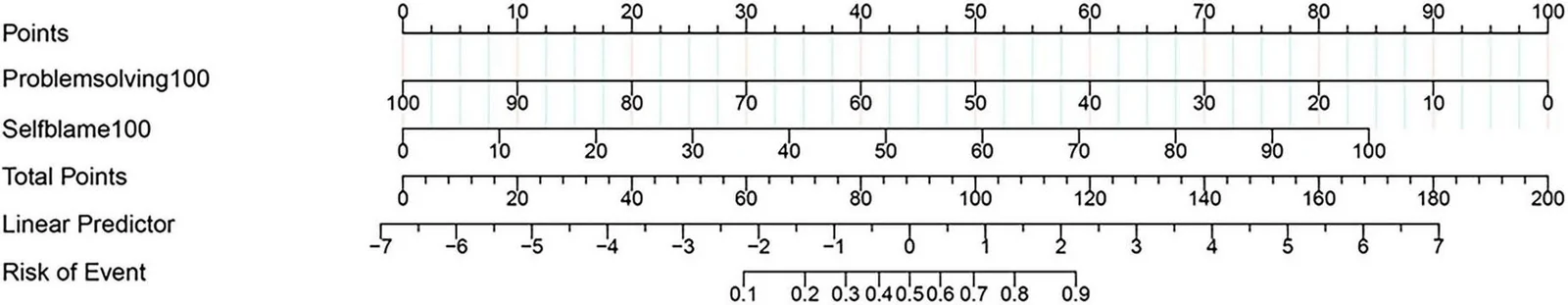
Nomogram for predicting the ride of non-suicidal self-injury (NSSI) among adolescents based on two independent coping style variables (problem-solving and self-blame).

**FIGURE 2:**
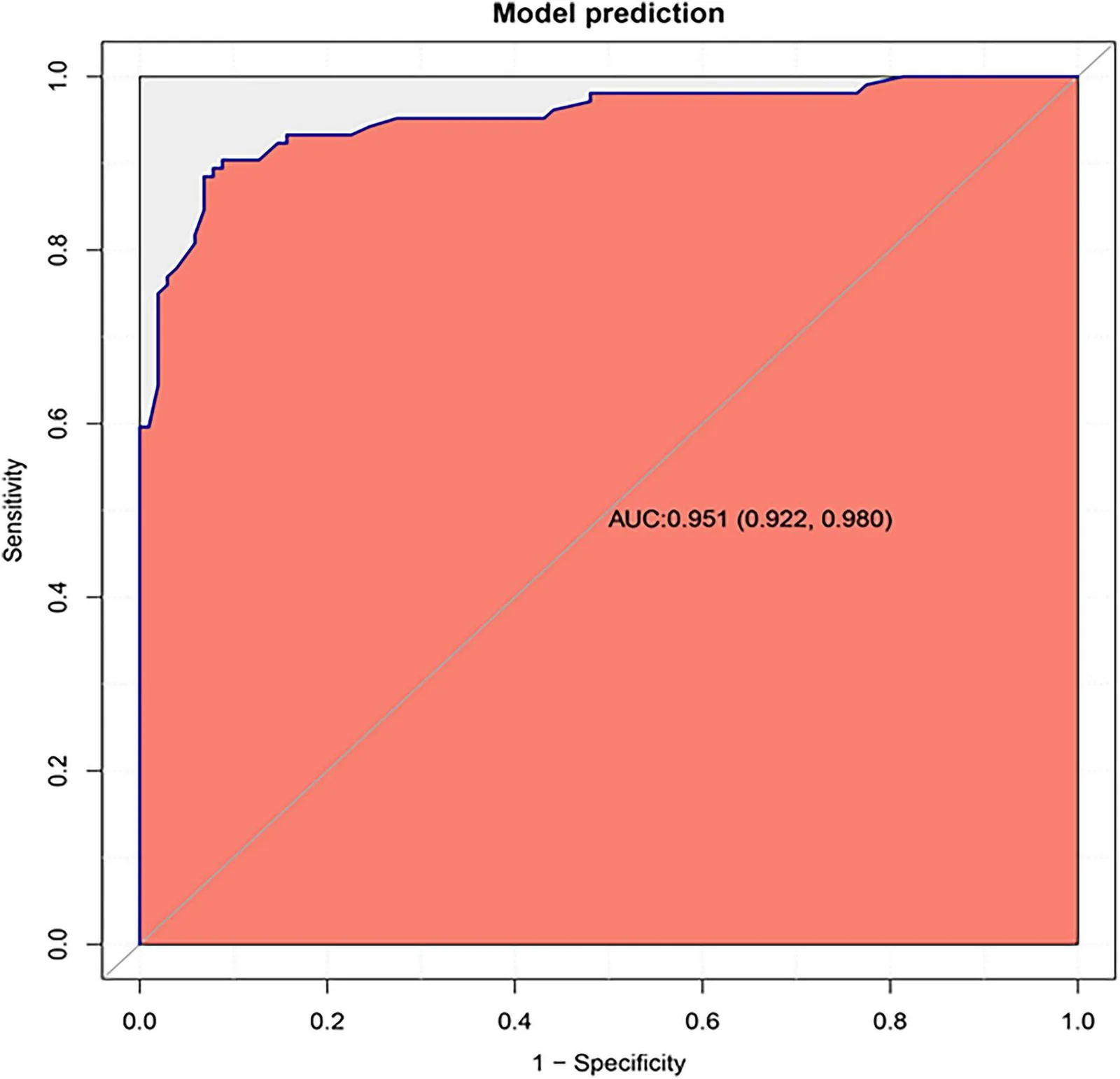
Receiver operating characteristic (ROC) curve of the prediction model for NSSI. The area under the curve (AUC) was 0.951 (95% CI 0.922–0.980), indicating excellent discriminative performance.

**FIGURE 3:**
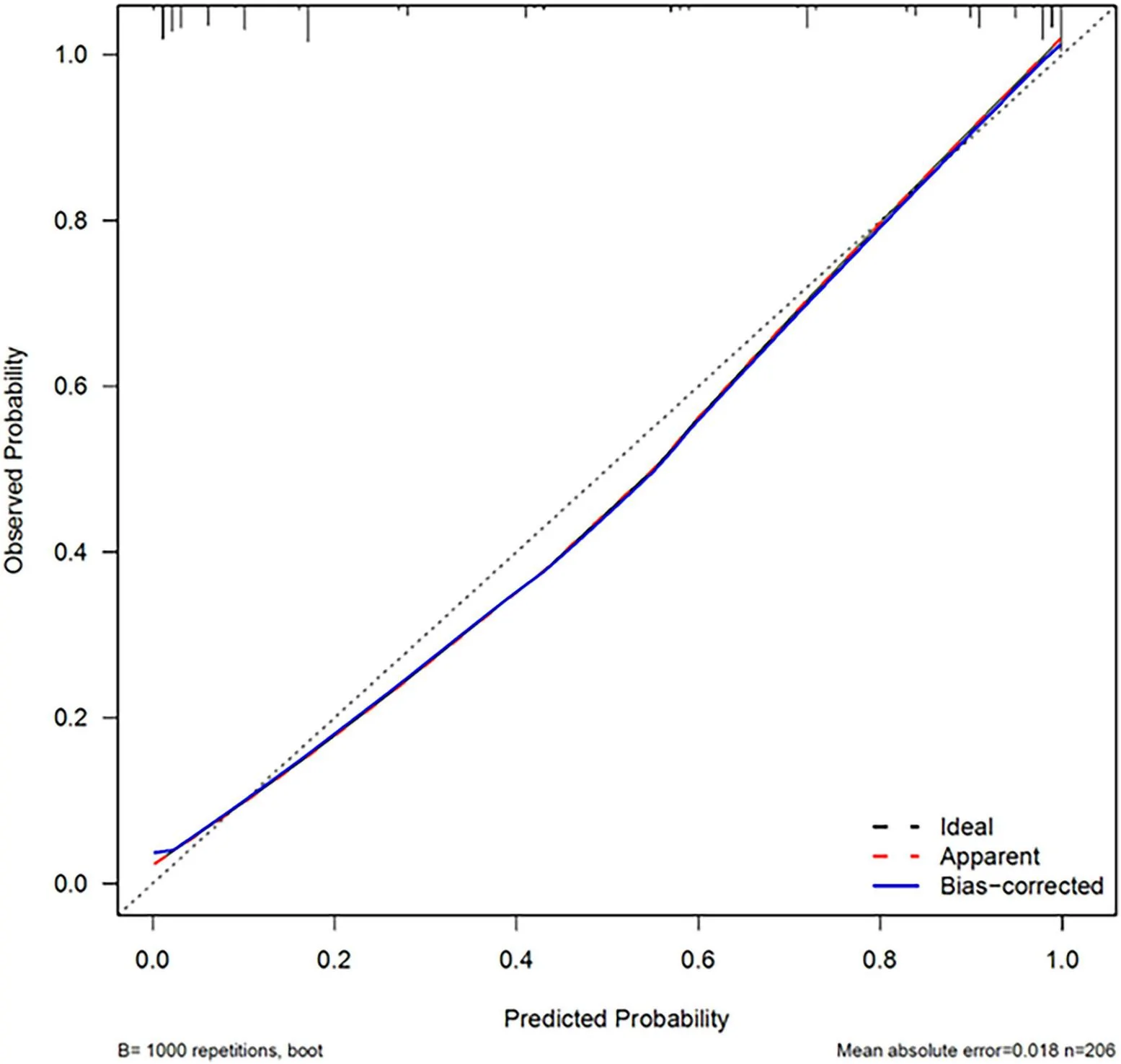
Calibration curve of the prediction model using 1000 bootstrap resamples. The bias-corrected curve matched the ideal line closely, with a mean absolute error (MAE) of 0.018, indicating good calibration.

**FIGURE 4:**
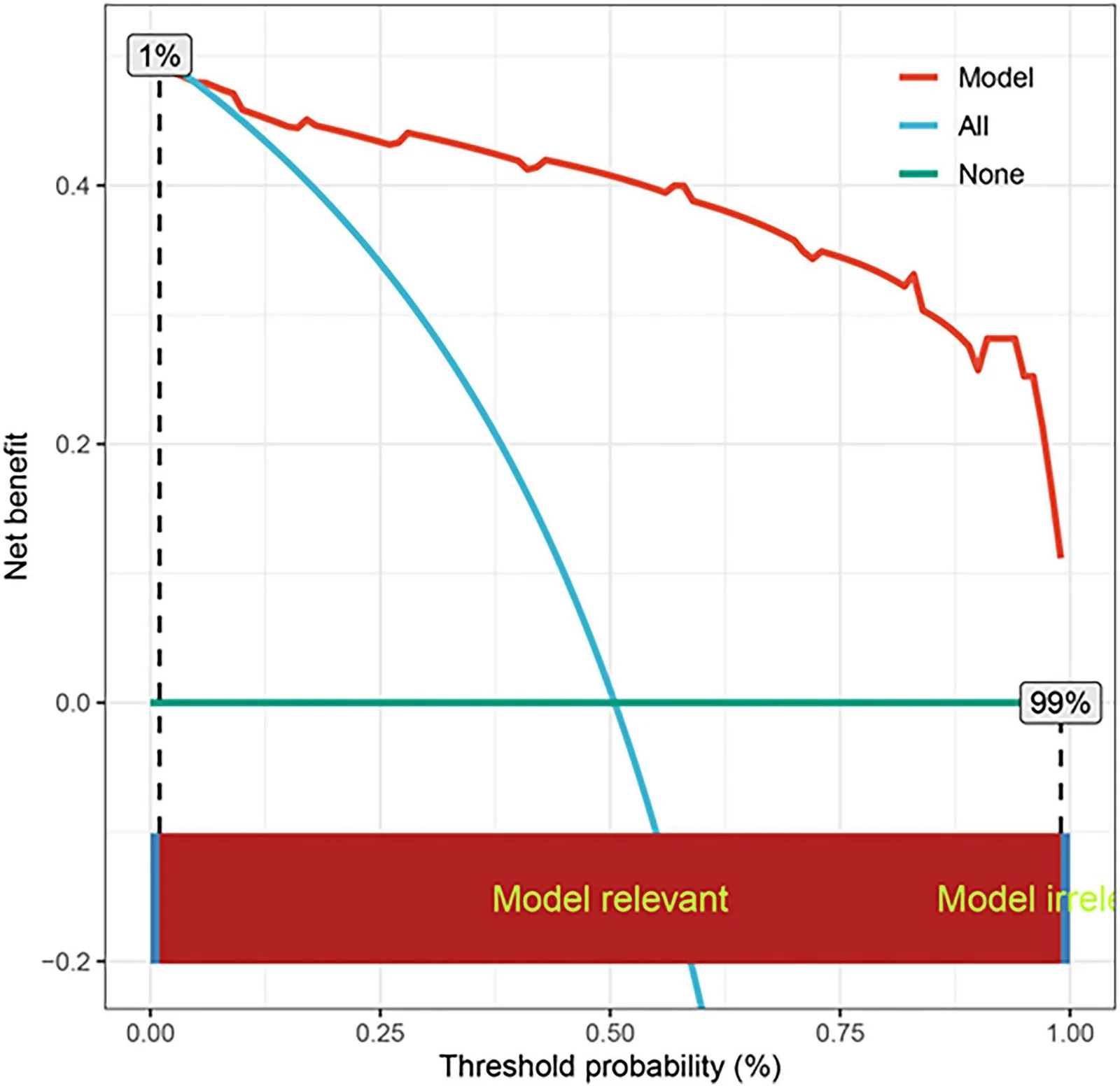
Decision curve analysis (DCA) of the nomogram for predicting NSSI ride among adolescents. The red line represents the net benefit of the nomogram model the blue line represents the “treat all” strategy, and the green line represents the “treat none” strategy.

## 4 Discussion

In terms of demographic characteristics, as hypothesized in the study, the NSSI group had a greater proportion of female participants, a higher rate of parental marital disruption (e.g., divorce), and poorer family economic status than the healthy controls group did. There may be several plausible explanations that account for the over-representation of girls in the NSSI group (Zhou et al., 2022). First, the existing epidemiological data indicate a higher prevalence of NSSI among girls (Tang et al., 2018), even though the underlying gender-specific mechanism for NSSI occurrence remains incompletely validated (Yang and Xin, 2018). Second, compared with males, female adolescents are more likely to self-report their mental-health symptoms and seek relevant services (Merikangas et al., 2011). Some boys with NSSI may be under-ascertained, as their self-injurious behaviors tend to be misattributed to attention-deficit/hyperactivity disorder (ADHD) or other disruptive-behavior disorders (Lustig et al., 2021). Third, NSSI behaviors such as self-cutting were measured mainly in this study. Girls tend to adopt cutting, scratching and pinching, whereas boys are more inclined to engage in other forms, including burning, head-banging and hair-pulling (Tang et al., 2018).

Indeed, no discrepancies with previous studies were observed for the psychosocial factors included in the intergroup analyses conducted in the present study, as adolescents with NSSI reported more childhood trauma experiences and higher levels of attachment-related insecurity than their healthy peers did. These results highlight the substantial influence of dysfunctional caregiver-infant relationships, early adverse experiences, and maladaptive coping on the development of NSSI. Most of the previous literature has suggested that childhood trauma experiences and insecure attachment patterns can lead directly to the occurrence of non-suicidal self-harm behaviors among adolescents (Eren Sarıkaya et al., 2024; Montanari et al., 2026). Exposure to adverse childhood experiences increases the risk of non-suicidal self-injury among adolescents with mental disorders (Liao et al., 2026). Assessment of trauma-related deficits in emotional processing and coping patterns is important when clinical evaluations and interventions for adolescents with NSSI behaviors are being conducted (Liu et al., 2026).

In this study, a logistic regression predictive model designed to identify independent psychological predictors of adolescent NSSI was developed and validated on the basis of attachment quality, childhood trauma, and coping style. The final multivariate model revealed two independent predictors: self-blame which is a definite risk factor, and problem-solving, which has a protective effect against adolescent NSSI. Notably, several of the coping style dimensions that differed between groups did not emerge as independent predictors in multivariate regression, which may be attributed to sample features and potential confounding effects among the variables. However, adaptive coping styles can mitigate the negative effects of these experiences (Zhou et al., 2022). We also highlight that future external validation datasets are needed to further evaluate model performance. These findings expand upon previous related research and provide a more comprehensive explanation of how coping styles contribute to self-injurious behavior among adolescents seeking clinical care (Liao et al., 2026). In line with our findings and previous literature, distant factors such as childhood trauma and attachment status may plausibly be linked to non-suicidal self-injury via individual coping styles, rather than exerting direct associations with NSSI. This represents a theoretical interpretation, which cannot be formally verified given our cross-sectional design and the absence of mediation analysis.

The results of this model indicate that self-blame, as a coping style, independently increases the risk of NSSI among adolescents, which is a finding that is consistent with the theoretical expectations of the self-punishment hypothesis. In accordance with this hypothesis, individuals may internalize early negative experiences such as childhood abuse into self-critical or self-deprecating cognitive patterns and subsequently use NSSI as a form of punitive self-harm (Glassman et al., 2007). For adolescents who are prone to self-blame, the physical pain that results from NSSI is often experienced as deserving or even purifying punishment, which serves to their alleviate feelings of inner shame, guilt, or self-loathing. Moreover, many self-injurers explicitly cite “self-punishment” as their primary motivation for engaging in NSSI (Nock and Prinstein, 2004). In our predictive model, self-blame retained its independent predictive power, which indicates that even after controlling for distal risk factors such as attachment styles and childhood trauma, this maladaptive cognitive coping strategy remained a proximal and key psychological mechanism triggering NSSI (Liu et al., 2026). Therefore, interventions that target adolescents with prominent tendencies toward self-blame should focus on the challenging and reconstructing of their irrational patterns of self-attribution.

Interestingly, problem solving, as a positive coping strategy, demonstrated a protective effect against NSSI in this study, which is an outcome strongly supported by the utility hypothesis. This hypothesis posits that NSSI becomes a common behavioral choice partly because it offers relatively quick and convenient emotional regulation without being constrained by environmental conditions, which makes it especially appealing to adolescents whose executive functions are not yet mature or who lack access to alcohol, drugs, or other resources (Nock, 2009). Conversely, adolescents who possess efficient and mature problem-solving skills have a superior alternative strategy. Individuals with strong problem-solving abilities are more likely to actively analyze difficulties, develop action plans, and mobilize available resources as a direct method of altering their stressors rather than as a passive endurance of distress or a means of relieving emotional discomfort. Adopting problem-focused coping styles and seeking social support may reduce the risk of NSSI among junior high school students (Zhou et al., 2022). This proactive coping mode significantly reduces the urgency of turning to NSSI as a temporary solution. Thus, higher scores on problem-solving in our nomogram corresponded to lower levels of NSSI risk (Peng et al., 2024), which indicates that enhancing adolescents’ ability to address real-life challenges can effectively replace the “practical function” served by NSSI, thereby reducing the likelihood of self-injury.

In this study, a prediction model for the risk of NSSI in adolescents was constructed on the basis of two statistically significant predictors, and the results were visualized using a nomogram to facilitate the discrimination of adverse events in clinical settings. The NSSI behavior itself is highly concealed. For example, skin injuries that are commonly found on parts of the body, such as the arms, are often deliberately concealed by adolescents. Moreover, some individuals may develop a similar addictive-like dependence on this behavior, which further exacerbates both physical and mental health damage (Cipriano et al., 2017; Nock, 2010). As the prevalence of NSSI behavior among adolescents increases, it may also lead to broader social functional problems, and thereby negatively affect the overall efficiency of social operations. These real risks highlight the need to implement case-control discrimination for adolescents who might engage in NSSI behavior in the future. Future work should explore whether the nomogram can help in the identification of adolescents who are potentially at risk of NSSI and inform targeted attention and intervention from both clinical and public health perspectives.

### 4.1 Strengths and practical implications

When built upon the two predictors (self-blame and problem-solving), the logistic-regression-derived nomogram achieves excellent discrimination, favorable calibration, and promising clinical net benefit. This simple tool supports self-injury risk discrimination for adolescents in clinical and school-based settings.

Although childhood trauma and insecure attachment did not independently predict NSSI in the multivariate model, multiple previous studies have demonstrated that these factors influence NSSI through coping-related mediating pathways. For adolescents who have been exposed to distal adversities such as childhood trauma, insecure attachment and adverse family backgrounds, psychological interventions rarely target these early-life risk factors directly.

Accordingly, modifying maladaptive coping patterns should be prioritized as a potential target for intervention among adolescents with NSSI. The nomogram serves as an exploratory differentiation instrument for early risk stratification among adolescents with potential NSSI risk. It enables clinicians to triage high-risk individuals and optimize mental-health resource allocation. External validation using independent cohorts is needed before routine clinical adoption.

### 4.2 Limitations

First, this study recruited participants from only a single city, which represents an important regional limitation. The sample was recruited from a single hospital and a single middle school, and discrepancies in unmeasured confounding characteristics between clinical adolescent patients and community-dwelling adolescents attending school may give rise to selection bias, which limits the clinical applicability of the findings, the nomogram primarily demonstrates discrimination between clinically identified NSSI cases and healthy controls. These results should be replicated in future research using community-based, multisite, and diverse samples.

Second, although the DSM-5 outlines six diagnostic criteria for NSSI, only frequency-based (NSSI-A) and functional-impact (NSSI-B) criteria were applied in the present study because of practical feasibility. Accordingly, our results may differ from those of studies that apply alternative sets of NSSI diagnostic criteria. Certain privacy-related psychosocial variables are highly sensitive. Some parents declined to participate because of concerns about interview privacy, which may introduce sampling bias. Even under anonymous survey conditions, some adolescents might withhold genuine responses out of fear that their true attitudes would be disclosed to their parents.

Third, methodologically, the cross-sectional design limits our ability to draw causal conclusions. The sample size was relatively modest, particularly in the multivariate logistic regression analysis that involved multiple predictors, and bootstrap resampling served only as internal validation. Accordingly, overly optimistic interpretations regarding clinical utility should be avoided. Prospective studies and independent external validation using alternate adolescent samples are needed before any clinical application can be considered.

## 5 Conclusion

Our findings support the self-punishment and pragmatic hypotheses: self-blame is associated with NSSI as a form of punishment that is perceived as deserved, whereas problem-solving serves as a protective factor by providing a more adaptive and “practical” alternative for emotion regulation. Importantly, distal risk factors such as childhood trauma and insecure attachment did not directly predict NSSI in the multivariate model, which implies that their effects are largely mediated by proximal coping patterns. This highlights the central role of coping styles in the psychological pathway leading early adversity to self-injurious behavior.

As a preliminary finding, the nomogram should be viewed solely as an exploratory differentiation tool. Its predictors are derived from adolescent baseline data and are not dependent on costly diagnostic equipment or substantial manpower-resource input, which may provide references for potential early-screening efforts. Its potential value for clinical practice remains to be further validated.

## Data Availability

The raw data supporting the conclusions of this article will be made available by the authors, without undue reservation.

## References

[B1] Adam K. S. Sheldon-Keller A. E. West M. (1996). Attachment organization and history of suicidal behavior in clinical adolescents. *J. Consult. Clin. Psychol.* 64, 264–272. 10.1037/0022-006x.64.2.264 8871410

[B2] Ainsworth M. Belhar M. Water E. Wall S. (1978). *Atterns of Attachment: A Psychological Study of the Strange Situation.* New York, NY: Psychology Press.

[B3] Alkema A. Marchi M. van der Zaag J. A. J. van der Sluis D. Warrier V. Ophoff R. A. et al. (2024). Childhood abuse v. neglect and risk for major psychiatric disorders. *Psychol. Med.* 54, 1598–1609. 10.1017/S0033291723003471 38018135

[B4] Ammerman B. A. Lengel G. J. Washburn J. J. (2021). Consideration of clinician and researcher opinions on the parameters of nonsuicidal self-injury disorder diagnostic criteria. *Psychiatry Res.* 296:113642. 10.1016/j.psychres.2020.113642 33352417

[B5] Battle D. E. (2013). Diagnostic and Statistical Manual of Mental Disorders (DSM). *Codas* 25, 191–192. 10.1590/s2317-17822013000200017 24413388

[B6] Bernstein D. P. Ahluvalia T. Pogge D. Handelsman L. (1997). Validity of the childhood trauma questionnaire in an adolescent psychiatric population. *J. Am. Acad. Child Adolesc. Psychiatry* 36, 340–348. 10.1097/00004583-199703000-00012 9055514

[B7] Bernstein D. P. Fink L. Handelsman L. Foote J. Lovejoy M. Wenzel K. et al. (1994). Initial reliability and validity of a new retrospective measure of child abuse and neglect. *Am. J. Psychiatry* 151, 1132–1136. 10.1176/ajp.151.8.1132 8037246

[B8] Bernstein D. P. Stein J. A. Newcomb M. D. Walker E. Pogge D. Ahluvalia T. et al. (2003). Development and validation of a brief screening version of the Childhood Trauma Questionnaire. *Child Abuse Negl.* 27, 169–190. 10.1016/s0145-2134(02)00541-0 12615092

[B9] Bowlby J. (1978). Attachment theory and its therapeutic implications. *Adolesc. Psychiatry* 6, 5–33. Avilable online at: https://pubmed.ncbi.nlm.nih.gov/742687/742687

[B10] Bowlby J. (1982). Attachment and loss: Retrospect and prospect. *Am. J. Orthopsychiatry* 52, 664–678. 10.1111/j.1939-0025.1982.tb01456.x 7148988

[B11] Brennan K. A. Shaver P. R. (1998). Attachment styles and personality disorders: Their connections to each other and to parental divorce, parental death, and perceptions of parental caregiving. *J. Pers.* 66, 835–878. 10.1111/1467-6494.00034 9802235

[B12] Chen Z. Li J. Liu J. Liu X. (2022). Adverse childhood experiences, recent negative life events, and non-suicidal self-injury among Chinese college students: The protective role of self-efficacy. *Child Adolesc. Psychiatry Ment. Health* 16:97. 10.1186/s13034-022-00535-1 36463208 PMC9719251

[B13] Cheng F. Shi L. Wang S. Jin Q. Xie H. Wang B. et al. (2023). The relationship between childhood traumatic experience and suicidal tendency in non-suicidal self-injury behavior patients. *BMC Psychiatry* 23:401. 10.1186/s12888-023-04863-0 37277735 PMC10240761

[B14] Cheng Y. Zhang P. Huang Y. Tang R. Zhang L. Sun J. et al. (2022). Construction and validation of a nomogram to predict overall survival in patients with breast sarcoma. *Front. Oncol.* 12:899018. 10.3389/fonc.2022.899018 36276138 PMC9582126

[B15] Cipriano A. Cella S. Cotrufo P. (2017). Nonsuicidal self-injury: A systematic review. *Front. Psychol.* 8:1946. 10.3389/fpsyg.2017.01946 29167651 PMC5682335

[B16] Cole-Detke H. Kobak R. (1996). Attachment processes in eating disorder and depression. *J. Consult. Clin. Psychol.* 64, 282–290. 10.1037//0022-006x.64.2.282 8871412

[B17] Collins N. L. (1996). Working models of attachment: Implications for explanation, emotion and behavior. *J. Pers. Soc. Psychol.* 71, 810–832. 10.1037//0022-3514.71.4.810 8888604

[B18] Eren Sarıkaya B. D. Ermiş Ç. Baykara H. B. Demirgören B. S. (2024). Non-suicidal self injury: Relationship with attachment, childhood trauma and dissociation. *Turk. Psikiyatri Derg.* 36, 1–7. 10.5080/u27163 39297252 PMC11987520

[B19] Estevez A. Jauregui P. Lopez-Gonzalez H. (2019). Attachment and behavioral addictions in adolescents: The mediating and moderating role of coping strategies. *Scand. J. Psychol.* 60, 348–360. 10.1111/sjop.12547 31087666

[B20] Glassman L. H. Weierich M. R. Hooley J. M. Deliberto T. L. Nock M. K. (2007). Child maltreatment, non-suicidal self-injury, and the mediating role of self-criticism. *Behav. Res. Ther.* 45, 2483–2490. 10.1016/j.brat.2007.04.002 17531192 PMC2034449

[B21] Gulbas L. E. Hausmann-Stabile C. De Luca S. M. Tyler T. R. Zayas L. H. (2015). An exploratory study of nonsuicidal self-injury and suicidal behaviors in adolescent Latinas. *Am. J. Orthopsychiatry* 85, 302–314. 10.1037/ort0000073 26052816 PMC4501885

[B22] Hamza C. A. Willoughby T. (2016). Nonsuicidal self-injury and suicidal risk among emerging adults. *J. Adolesc. Health* 59, 411–415. 10.1016/j.jadohealth.2016.05.019 27485906

[B23] He Q. Wu Y. J. Wang Y. C. (2026). Adverse childhood experiences and the risk of non-suicidal self-injury: A meta-analysis. *Front. Psychiatry* 17:1790171. 10.3389/fpsyt.2026.1790171 42052543 PMC13111225

[B24] Kiekens G. Claes L. Demyttenaere K. Auerbach R. P. Green J. G. Kessler R. C. et al. (2016). Lifetime and 12-Month nonsuicidal self-injury and academic performance in college freshmen. *Suicide Life Threat Behav.* 46, 563–576. 10.1111/sltb.12237 26954061

[B25] Kiekens G. Claes L. Hasking P. Mortier P. Bootsma E. Boyes M. et al. (2023). A longitudinal investigation of non-suicidal self-injury persistence patterns, risk factors, and clinical outcomes during the college period. *Psychol. Med.* 53, 6011–6026. 10.1017/S0033291722003178 36325723

[B26] Klonsky E. D. Victor S. E. Saffer B. Y. (2014). Nonsuicidal self-injury: What we know, and what we need to know. *Can. J. Psychiatry* 59, 565–568. 10.1177/070674371405901101 25565471 PMC4244874

[B27] Lengel G. J. Mullins-Sweatt S. N. (2013). Nonsuicidal self-injury disorder: Clinician and expert ratings. *Psychiatry Res.* 210, 940–944. 10.1016/j.psychres.2013.08.047 24045137

[B28] Liao L. Jian Y. Li J. J. Zhang D. N. Meng W. Shi Z. et al. (2026). Effect on adverse childhood experiences and non-suicidal self-injury in adolescents with mental illness: The mediation effect of coping styles. *Child Abuse Negl.* 173:107890. 10.1016/j.chiabu.2026.107890 41638178

[B29] Liu Y. Liu Y. Zhou R. Hou C. (2026). The relationship between childhood trauma and non-suicidal self-injury in adolescents with depressive disorders: The mediating role of alexithymia and coping strategies. *Front. Psychiatry* 17:1744863. 10.3389/fpsyt.2026.1744863 41908130 PMC13021842

[B30] Lustig S. Koenig J. Resch F. Kaess M. (2021). Help-seeking duration in adolescents with suicidal behavior and non-suicidal self-injury. *J. Psychiatr. Res.* 140, 60–67. 10.1016/j.jpsychires.2021.05.037 34098387

[B31] Manca M. Presaghi F. Cerutti R. (2014). Clinical specificity of acute versus chronic self-injury: Measurement and evaluation of repetitive non-suicidal self-injury. *Psychiatry Res.* 215, 111–119. 10.1016/j.psychres.2013.10.010 24210667

[B32] Merikangas K. R. He J. P. Burstein M. Swendsen J. Avenevoli S. Case B. et al. (2011). Service utilization for lifetime mental disorders in U.S. adolescents: Results of the National Comorbidity Survey-Adolescent Supplement (NCS-A). *J. Am. Acad. Child Adolesc. Psychiatry* 50, 32–45. 10.1016/j.jaac.2010.10.006 21156268 PMC4408275

[B33] Michelson D. Bhugra D. (2012). Family environment, expressed emotion and adolescent self-harm: A review of conceptual, empirical, cross-cultural and clinical perspectives. *Int. Rev. Psychiatry* 24, 106–114. 10.3109/09540261.2012.657613 22515458

[B34] Mikulincer M. Shaver P. R. (2012). An attachment perspective on psychopathology. *World Psychiatry* 11, 11–15. 10.1016/j.wpsyc.2012.01.003 22294997 PMC3266769

[B35] Montanari S. Callovini T. Bartolucci G. Marcelli I. Autullo G. Koukopoulos A. et al. (2026). Childhood trauma assessed using the childhood trauma questionnaire and its influence on pediatric mood disorders and self-harm: A systematic review and meta-analysis. *Child Abuse Negl.* 175:107979. 10.1016/j.chiabu.2026.107979 41806686

[B36] Moran P. Chandler A. Dudgeon P. Kirtley O. J. Knipe D. Pirkis J. et al. (2024). The Lancet commission on self-harm. *Lancet* 404, 1445–1492. 10.1016/S0140-6736(24)01121-8 39395434

[B37] Nock M. K. (2009). Why do people hurt themselves? new insights into the nature and functions of self-injury. *Curr. Dir. Psychol. Sci.* 18, 78–83. 10.1111/j.1467-8721.2009.01613.x 20161092 PMC2744421

[B38] Nock M. K. (2010). Self-injury. *Annu. Rev. Clin. Psychol.* 6, 339–363. 10.1146/annurev.clinpsy.121208.131258 20192787

[B39] Nock M. K. Prinstein M. J. (2004). A functional approach to the assessment of self-mutilative behavior. *J. Consult. Clin. Psychol.* 72, 885–890. 10.1037/0022-006X.72.5.885 15482046

[B40] Pascuzzo K. Cyr C. Moss E. (2013). Longitudinal association between adolescent attachment, adult romantic attachment, and emotion regulation strategies. *Attach. Hum. Dev.* 15, 83–103. 10.1080/14616734.2013.745713 23216393

[B41] Peng M. Zhang L. Wu Q. Liu H. Zhou X. Cheng N. et al. (2024). The effects of childhood trauma on nonsuicidal self-injury and depressive severity among adolescents with major depressive disorder: The different mediating roles of positive and negative coping styles. *J. Affect. Disord.* 361, 508–514. 10.1016/j.jad.2024.06.037 38909757

[B42] Qu D. Wen X. Liu B. Zhang X. He Y. Chen D. et al. (2023). Non-suicidal self-injury in Chinese population: A scoping review of prevalence, method, risk factors and preventive interventions. *Lancet Reg. Health West Pac.* 37:100794. 10.1016/j.lanwpc.2023.100794 37693882 PMC10485683

[B43] Robinson K. Garisch J. A. Kingi T. Brocklesby M. O’Connell A. Langlands R. L. et al. (2019). Reciprocal risk: The longitudinal relationship between emotion regulation and non-suicidal self-injury in adolescents. *J. Abnorm. Child Psychol.* 47, 325–332. 10.1007/s10802-018-0450-6 29923162

[B44] Shapiro D. L. Levendosky A. A. (1999). Adolescent survivors of childhood sexual abuse: The mediating role of attachment style and coping in psychological and interpersonal functioning. *Child Abuse Negl.* 23, 1175–1191. 10.1016/s0145-2134(99)00085-x 10604070

[B45] Tang J. Li G. Chen B. Huang Z. Zhang Y. Chang H. et al. (2018). Prevalence of and risk factors for non-suicidal self-injury in rural China: Results from a nationwide survey in China. *J. Affect. Disord.* 226, 188–195. 10.1016/j.jad.2017.09.051 28988001

[B46] Tao Y. Bi X. Deng M. (2020). The impact of parent–child attachment on self-injury behavior: Negative emotion and emotional coping style as serial mediators. *Front. Psychol.* 11:1477. 10.3389/fpsyg.2020.01477 32848972 PMC7411466

[B47] Tasca G. A. Szadkowski L. Illing V. Trinneer A. Grenon R. Demidenko N. et al. (2009). Adult attachment, depression, and eating disorder symptoms: The mediating role of affect regulation strategies. *Personal. Individ. Differ.* 47, 662–667. 10.1016/j.paid.2009.06.006

[B48] Woo J. Wrath A. J. Adams G. C. (2022). The relationship between attachment and self-injurious behaviors in the child and adolescent population: A systematic review of the literature. *Arch. Suicide Res.* 26, 406–427. 10.1080/13811118.2020.1804024 32780672

[B49] Xiao Jihua X. X. (1996). Reliability and validity of the coping style questionnaire. *Chinese Mental Health J.* 4, 164–168. Available online at: https://zxws.cbpt.cnki.net/portal/journal/portal/client/paper/0506e12721c45f4e61a4ec1ea0ec7508

[B50] Yang X. Xin M. (2018). “Boy Crisis” or “Girl Risk”? The gender difference in nonsuicidal self-injurious behavior among middle-school students in china and its relationship to gender role conflict and violent experiences. *Am. J. Mens. Health* 12, 1275–1285. 10.1177/1557988318763522 29577836 PMC6142131

[B51] Zhang Q. Hou Z. J. Fraley R. C. Hu Y. Zhang X. Zhang J. et al. (2022). Validating the experiences in close relationships-relationship structures scale among chinese children and adolescents. *J. Pers. Assess.* 104, 347–358. 10.1080/00223891.2021.1947844 34292844

[B52] Zhou J. Zhang J. Huang Y. Zhao J. Xiao Y. Zhang S. et al. (2022). Associations between coping styles, gender, their interaction and non-suicidal self-injury among middle school students in rural west China: A multicentre cross-sectional study. *Front. Psychiatry* 13:861917. 10.3389/fpsyt.2022.861917 36016979 PMC9395723

